# Improving the SIENA performance using BEaST brain extraction

**DOI:** 10.1371/journal.pone.0196945

**Published:** 2018-09-20

**Authors:** Kunio Nakamura, Simon F. Eskildsen, Sridar Narayanan, Douglas L. Arnold, D. Louis Collins, The Alzheimer's Disease Neuroimaging Initiative

**Affiliations:** 1 McConnell Brain Imaging Centre, Montreal Neurological Institute, McGill University, Montreal, Quebec, Canada; 2 Department of Biomedical Engineering, Lerner Research Institute, Cleveland Clinic, Cleveland, Ohio, United States of America; 3 Centre of Functionally Integrative Neuroscience, Aarhus University, Aarhus, Denmark; 4 NeuroRx Research, Montreal, Quebec, Canada; NIH Clinical Center, UNITED STATES

## Abstract

We present an improved image analysis pipeline to detect the percent brain volume change (PBVC) using SIENA (Structural Image Evaluation, using Normalization, of Atrophy) in populations with Alzheimer’s dementia. Our proposed approach uses the improved brain extraction mask from BEaST (Brain Extraction based on nonlocal Segmentation Technique) instead of the conventional BET (Brain Extraction Tool) for SIENA. We compared four varying options of BET as well as BEaST and applied these five methods to analyze scan-rescan MRIs in ADNI from 332 subjects, longitudinal ADNI MRIs from the same 332 subjects, their repeat scans over time, and OASIS longitudinal MRIs from 123 subjects. The results showed that BEaST brain masks were consistent in scan-rescan reproducibility. The cross-sectional scan-rescan error in the absolute percent brain volume difference measured by SIENA was smallest (*p*≤0.0187) with the proposed *BEaST-SIENA*. We evaluated the statistical power in terms of effect size, and the best performance was achieved with *BEaST-SIENA* (1.2789 for ADNI and 1.095 for OASIS). The absolute difference in PBVC between scan-dataset (volume change from baseline to year-1) and rescan-dataset (volume change from baseline repeat scan to year-1 repeat scan) was also the smallest with *BEaST-SIENA* compared to the BET-based SIENA and had the highest correlation when compared to the BET-based SIENA variants. In conclusion, our study shows that BEaST was robust in terms of reproducibility and consistency and that SIENA’s reproducibility and statistical power are improved in multiple datasets when used in combination with BEaST.

## Introduction

Chronic brain atrophy is one of the pathologic hallmarks in neurological diseases such as multiple sclerosis and Alzheimer’s disease (AD). Advanced image processing of magnetic resonance imaging (MRI) allows *in vivo* quantification of brain atrophy. Of several sophisticated quantification algorithms available, SIENA (Structural Image Evaluation, using Normalization, of Atrophy) [1] from the FSL package [2], that has seen particularly wide use in multiple sclerosis research and clinical trials. The major advantages of the SIENA method are 1) skull-constrained registration, which corrects for incorrect pixel sizes [1] and reduces distortion artifacts [3], 2) halfway-space transformation whereby the images are equally interpolated, 3) measurement of edge shift using the first derivatives of the intensity profiles at the brain edge for sub-voxel accuracy, and 4) self-calibration to determine the ratio between surface area and volume to measure the percent volume change. SIENA is robust for different MRI slice thicknesses, has low transitivity error [1], and is statistically powerful [4]. SIENA has been used in many centers for research studies and clinical trials with varying protocols. On the other hand, SIENA is not commonly used in AD studies. One possible reason may be that some patients with AD exhibit extreme atrophy, and the FSL tools may not perform optimally in such cases. In fact, our previous work has shown that the performance of brain extraction in BET/FSL [5] worsened when applied to ADNI data compared to data from young normal subjects in the ICBM dataset in terms of Dice similarity coefficient (0.944 and 0.975), false positive rate (3.81 and 1.28), and false negative rate (2.71 and 0.45) [6]. In the same study, we demonstrated that the new method called BEaST (Brain Extraction based on nonlocal Segmentation Technique) improved the accuracy of brain extraction. The use of SIENA in AD studies may bridge these neurological conditions (such as multiple sclerosis and Alzheimer’s disease) and allow meta-analysis to compare or merge such datasets in the future. In the current study, we investigated the effect on SIENA measurements when applied in conjunction with BEaST-extracted brain masks. We 1) applied SIENA using various brain extraction approaches including BET and BEaST; 2) compared the extracted brain masks by measuring the overlaps between subjects and consistency within subjects; 3) compared SIENA scan-rescan reproducibility, and finally 4) compared the statistical power in terms of effect size and required sample size to detect significant changes using SIENA.

## Materials and methods

### Dataset

#### ADNI

We downloaded “Original” images from ADNI datasets (S1). These images are not pre-processed and meant to better represent typical images in clinical and research studies where B1- or distortion- corrections are not performed. The inclusion criteria were: Field strength = 1.5-Tesla, Slice Thickness = 0.5–1.9 mm, Weighting = T1, Image Description = MP-RAGE, Subject Group = AD or normal control (NC), Visits = complete set of baseline, repeat baseline, and month 12. Cases with severe image artifacts were removed (n = 1). The remaining 139 AD patients (mean age (SD) = 75.2 (7.5) years; male proportion = 52%) and 193 normal controls (mean age (SD) = 77.2 (5.5) years; male proportion = 52%) were analyzed for percent brain volume change between baseline and year 1 and between baseline and repeat baseline. For each baseline scan, we also downloaded the repeat scan acquired on the same day. The MRI sequence is described elsewhere [7]

#### OASIS

We also used the OASIS (Open Access Series of Imaging Studies) Longitudinal MRI Data in Nondemented and Demented Older Adults (OASIS2) [8]. We selected the patients who had the follow-up MRI between 365–1095 days (1–3 years). At each time-point in the OASIS study, 4 MP-RAGE scans were acquired and averaged together. Since typical studies would only acquire one MP-RAGE per time-point, we only used the first image for the study presented here. One subject was dropped due to an image inconsistency. Thus, the sample comprised 59 demented subjects (mean age (SD) = 74.9 (6.9) years; male proportion = 54%) and 64 non-demented subjects (mean age (SD) = 75.6 (8.6) years; male proportion = 30%).

### Pre-processing

All images were converted to MINC format (Medical Imaging NetCDF, Montreal Neurological Institute, McGill University, Montreal, Quebec, Canada, http://www.mcgill.ca/bic/software-atlases/minc). All the pre-processing was performed using minc-toolkit (ITK4v2 branch, https://github.com/BIC-MNI/minc-toolkit). The pre-processing was similar to previous studies [9–11] and consisted of the following: 1) N3 correction [12], 2) de-noising using non-local means [13], 3) linear affine standard space registration [14] to nonlinear ICBM atlas (ICBM 2009c [15]), 4) intensity normalization based on histogram-matching, 5) brain extraction using BEaST (version 1.15, http://www.bic.mni.mcgill.ca/BEaST) [6], and 6) refinement of linear affine registration, N3 correction, and intensity normalization using the BEaST brain extraction mask. Importantly, the input to BEaST was N3-corrected, de-noised, and intensity-normalized image, resampled in standard space using 12-parameter affine registration. The BEaST library consisted of 10 samples from ICBM library [16], 10 samples from NIH study of normal brain development [17], and 60 samples from ADNI as described in the original study [6]. We left-right flipped the template images along the x-axis to artificially increase the sample for a total of 160 samples in the BEaST library. Of the 160 library images, BEaST preselects the 20 best matching samples for processing each image independently. The highest processing resolution of BEaST was 2mm with median filter set as a post-processing step. The mask was then brought back from standard space to the subject’s native space and resampled to the original resolution. Of 323 ADNI subjects, 39 cases were overlapping with images included in the library. For these 39 cases, we excluded the case from the library to avoid the potential advantage for BEaST.

### SIENA

**Fig 1:**
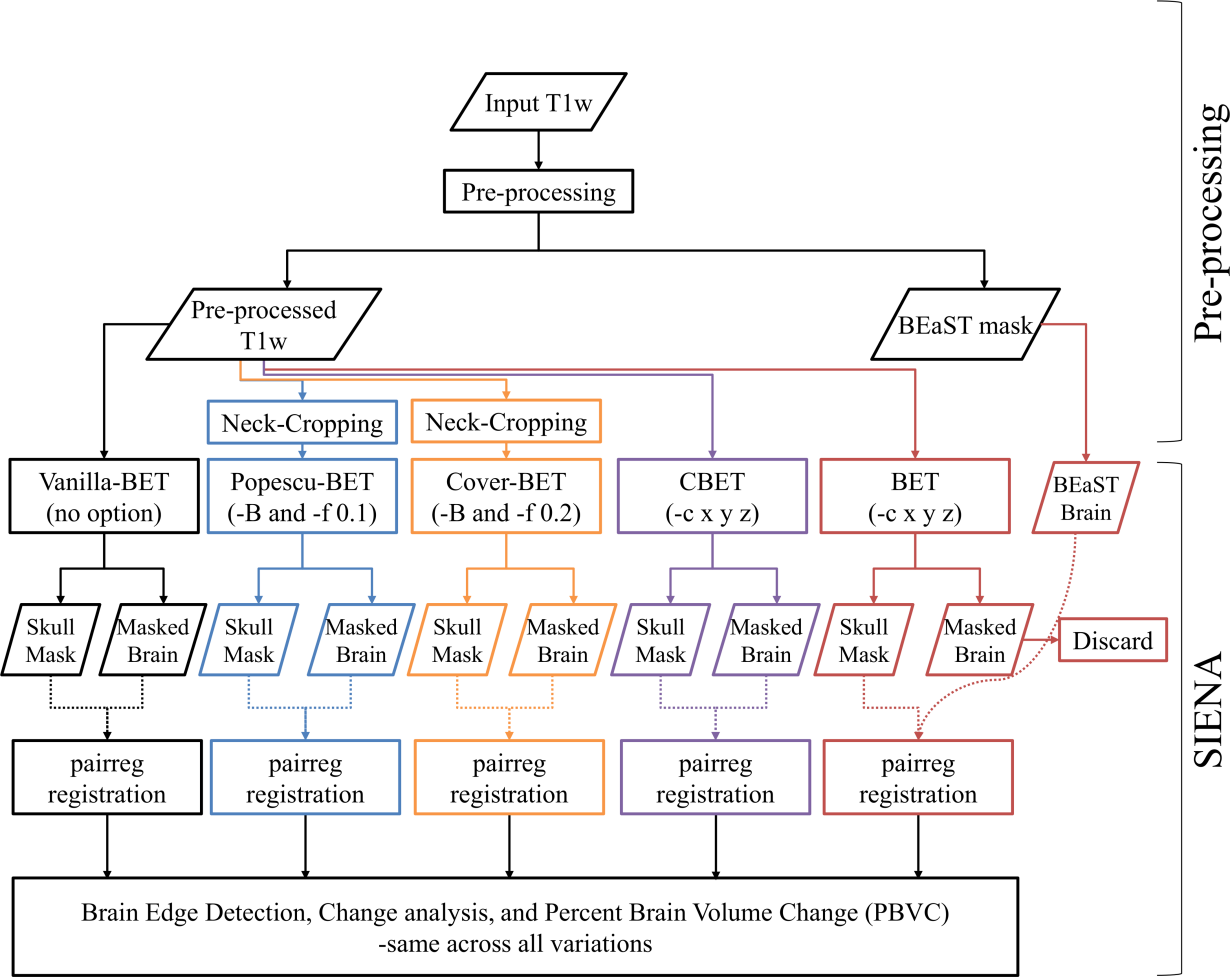
Flowchart diagram of the processing pipelines: The BET brain masks and masked brain images are discarded for the BEaST pipeline and replaced by BEaST brain masks and masked images.

We used SIENA (FSL version 5.0, https://fsl.fmrib.ox.ac.uk/fsl/) on CentOS 6.3 on the supercomputer Guillimin from McGill University (http://www.hpc.mcgill.ca). We measured the percent brain volume change (PBVC) in three ways: (1) between the baseline scan and follow-up (year-1) (original PBVC), (2) between baseline scan and baseline repeat scan for cross-sectional scan-rescan comparison, and (3) between repeat-scan at baseline and repeat-scan at year 1 (repeat PBVC) for longitudinal scan-rescan comparison. We compared five variations of SIENA as described next and summarized in **Table 1** and **Fig 1**. We ran the analysis completely automatically.

**Table 1:** Summary of 5variations of SIENA.

Method	BET options	SIENA option	Neck Cropping	Brain Extraction
**Vanilla-SIENA**	None	-m	None	BET
**Popescu-SIENA**	-B -f 0.1	-m	Yes	BET
**Cover-SIENA**	-B -f 0.2	-m	Yes	BET
**CBET-SIENA**	-c <x y z>	-m	None	BET
**BEaST-SIENA**	-c <x y z>	-m	None	BEaST

#### 1. Vanilla-SIENA

As a comparator, we applied the SIENA with “-m” option, which performs standard-space masking as well as BET, as a previous study has shown the benefit of standard space masking [18]. While typical research centers do not perform *Vanilla-SIENA*, this version serves as a traditional reference, and less advanced centers still use this option.

#### 2. Popescu-SIENA

To compare an improved brain extraction method within the FSL framework, we applied the options suggested by Popescu et al. [19]. In their study, different BET options were compared with respect to the manual segmentation using multi-center MRIs from multiple sclerosis patients. As described, we performed the neck-cropping using the field-of-view region-of-interest (ROI) created in MNI space. We also used the BET [5] options “-B” and “-f 0.1,” where “-B” corrects the bias field and cleans up the neck and “-f” is a fractional intensity threshold that would lead to a larger brain outline at the bottom with smaller f-value.

#### 3. Cover-SIENA

We also applied the options suggested by Cover et al. [20]. In that study, “-B -f 0.2” appears to be the optimal parameter for analysis of 1.5-Tesla MRIs in ADNI. We used the same neck-cropping as in *Popescu-SIENA* and changed the BET option. The only difference between *Popescu-SIENA* was the f-option.

#### 4. CBET-SIENA

We used an option “-c” to define the center of gravity, which was calculated from a predefined coordinate in the standard ICBM space (0,-12,4) between left and right thalami and the affine standard-space registration matrix used to map the MRI into standard space. There was no neck-cropping. There were no additional options for BET. This version serves as a reference to *BEaST-SIENA*.

#### 5. BEaST- SIENA

In the proposed pipeline, we replaced the BET brain mask with the BEaST brain mask. BEaST was previously validated in terms of accuracy to be superior to BET and the VBM8 toolbox of SPM8 [6]. In this pipeline, we ran BET only to produce the skull mask since SIENA requires the skull mask for registration. We again used the “-c” option to set the center of gravity, using the same coordinate as in *CBET-SIENA*. There was no neck-cropping.

### Assessment of methods

#### Mask comparison

To detect gross brain masking errors, we generated average masks in standard space using the following procedure: we 1) nonlinearly registered all baseline MR images to the ICBM 2009c template using ANTS (http://stnava.github.io/ANTs) [21], 2) transformed and resampled the brain masks from the native space to standard space using the transformation from ANTS, 3) computed the voxel-by-voxel mean and standard deviation of the masks from each subject for each time-point, and 4) repeated the process for each of the five SIENA variations (Vanilla, Popescu, Cover, CBET, and BEaST). ANTS was applied to the full image without brain extraction. We did not use the brain mask to avoid introducing the mask’s influence on the performance of nonlinear registration. Note that BEaST was applied independently for baseline and follow-up image. In other words, there was no affine registration between baseline and follow-up scans in generating BEaST masks so that the comparison of masks is fair across the pipelines. We visually inspected the general accuracy of nonlinear registration. This test was only intended to show *gross* errors in brain extraction since there is no gold standard for each subject, and since the definition of true brain extraction may be different for BET and BEaST. We also counted the number of failures, defined by “incomplete analysis” whereby SIENA did not produce a valid value, which happened due to poor brain extraction and/or poor registration.

#### Cross-sectional scan-rescan error

First, the reproducibility of brain extracted masks was compared among BEaST and the four BET variations using the cross-sectional ADNI scan-rescan data acquired on the same day. We calculated absolute volume difference, sensitivity, specificity, Jaccard similarity index, and mean surface distance between the scan and rescan brain extraction masks. All measurements were made in native space with linear rigid-body registration without the use of brain extraction masks. Thus, we used the same linear registration matrix for all 5 versions. We also created absolute difference maps between the scan and rescan brain masks for each subject and averaged them in the nonlinearly resampled standard space for each SIENA method.

To evaluate the impact on reproducibility of SIENA, we measured the percent brain volume difference and its absolute value for each of the 5 variations of SIENA.

#### Effect size and sample size

To evaluate the quantitative improvement on statistical power, we calculated the effect size to detect a 25% change in PBVC over a 12-month period for each method, for each dataset. For ADNI, PBVC was measured between baseline scan and year-1 scan (original PBVC). The OASIS PBVC values were annualized since the MRI intervals were not consistent across subjects. The effect size was calculated using the standard equation as follows:

$$Effect\;Size=\frac{\mu_{NC}-\mu_{AD}}{\sqrt{\frac{(n_{NC}-1)\sigma_{NC}^{2}+(n_{AD}-1)\sigma_{AD}^{2}}{n_{NC}+n_{AD}-2}}}$$ Eq 1

where the denominator is a pooled standard deviation, σ is the group PBVC standard deviation, and μ is the group PBVC mean obtained from longitudinal data. This equation includes the effect of normal aging thus the hypothesized treatment effect acts on the difference between AD and healthy controls; that is, a 100% treatment effect leads to patients having the same atrophy rate as healthy controls. We then calculated the required sample size per arm from the effect size according to the Eq 2:

$$N=Ceil\;\left(\frac{2{{\left({SD}_{p}\right)}^{2}(u+v)}^{2}}{{\left(TxE(\mu_{AD}-\mu_{Normal})\right)}^{2}}\right)=Ceil\;\left(\frac{2{\left(u+v\right)}^{2}}{{\left(\left(TxE)(Effect\;Size\right)\right)}^{2}}\right)$$ Eq 2

where *SD*_*p*_ is the pooled standard deviation, *u* = 0.842 for 80% power, *v* = 1.96 to test at 0.05 significance level, Ceil is the round-up operator, and *TxE* is the predefined treatment effect (25%), which is assumed to take immediate and constant effect. All available PBVC values were included in the analysis.

#### Longitudinal scan-rescan error

Using the ADNI rescan data acquired over time, we measured PBVC between the baseline repeat and year-1 repeat (repeat PBVC). We compared the repeat PBVC with the original PBVC between baseline scan and year-1 scan and calculated the mean difference and mean absolute difference in PBVC as well as their correlation and regression (slope and intercept).

## Results

**Fig 2:**
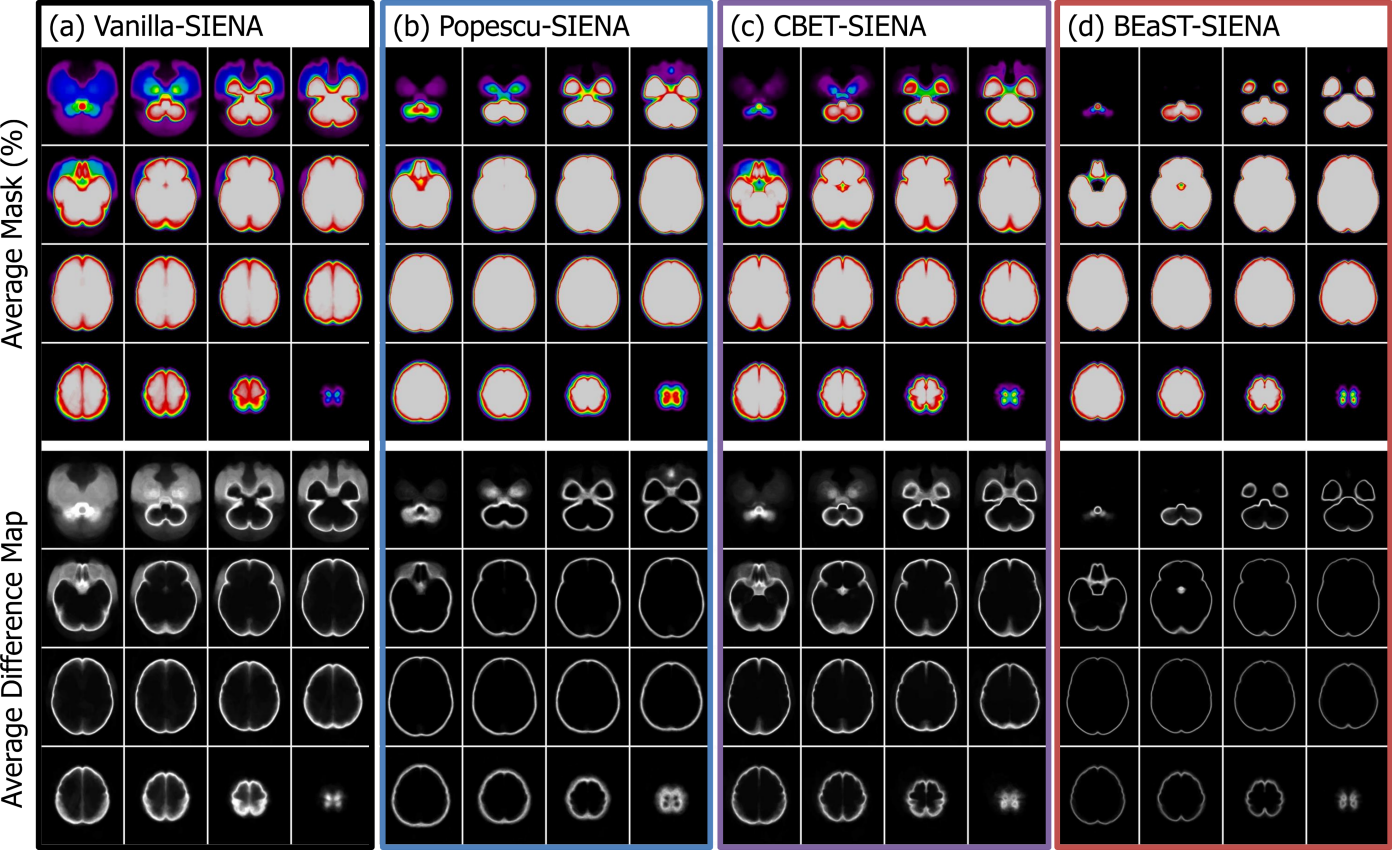
Top four rows: twelve transverse slices through the voxel-by-voxel average of 910 masks generated for each method (from left to right: *Vanilla-SIENA*, *Popescu-SIENA*, *Cover-SIENA*, *CBET-SIENA*, and *BEaST-SIENA*), on spectral colormap. The white and black are the areas with complete agreement while colored voxels show variable degree of disagreement shown on the colorbar. BEaST masks overall show the smallest variability compared to other methods. Bottom four rows: The average difference map from scan-rescan dataset for each method. The difference map was created in the native space using SIENA’s flirt registration matrix. Then the difference maps were nonlinearly transformed into the common ICBM space for averaging. The range for all difference maps was set to a constant; note the darker intensity for BEaST, indicating smaller error.

**Fig 2** shows the qualitative evaluations of methods. BEaST appears to have the smallest variability in average mask obtained from 910 masks in 332 ADNI and 123 OASIS cases (top). BEaST also had consistent segmentation within subjects from the scan-rescan pairs from 332 ADNI cases acquired on the same day (bottom).

**Fig 3:**
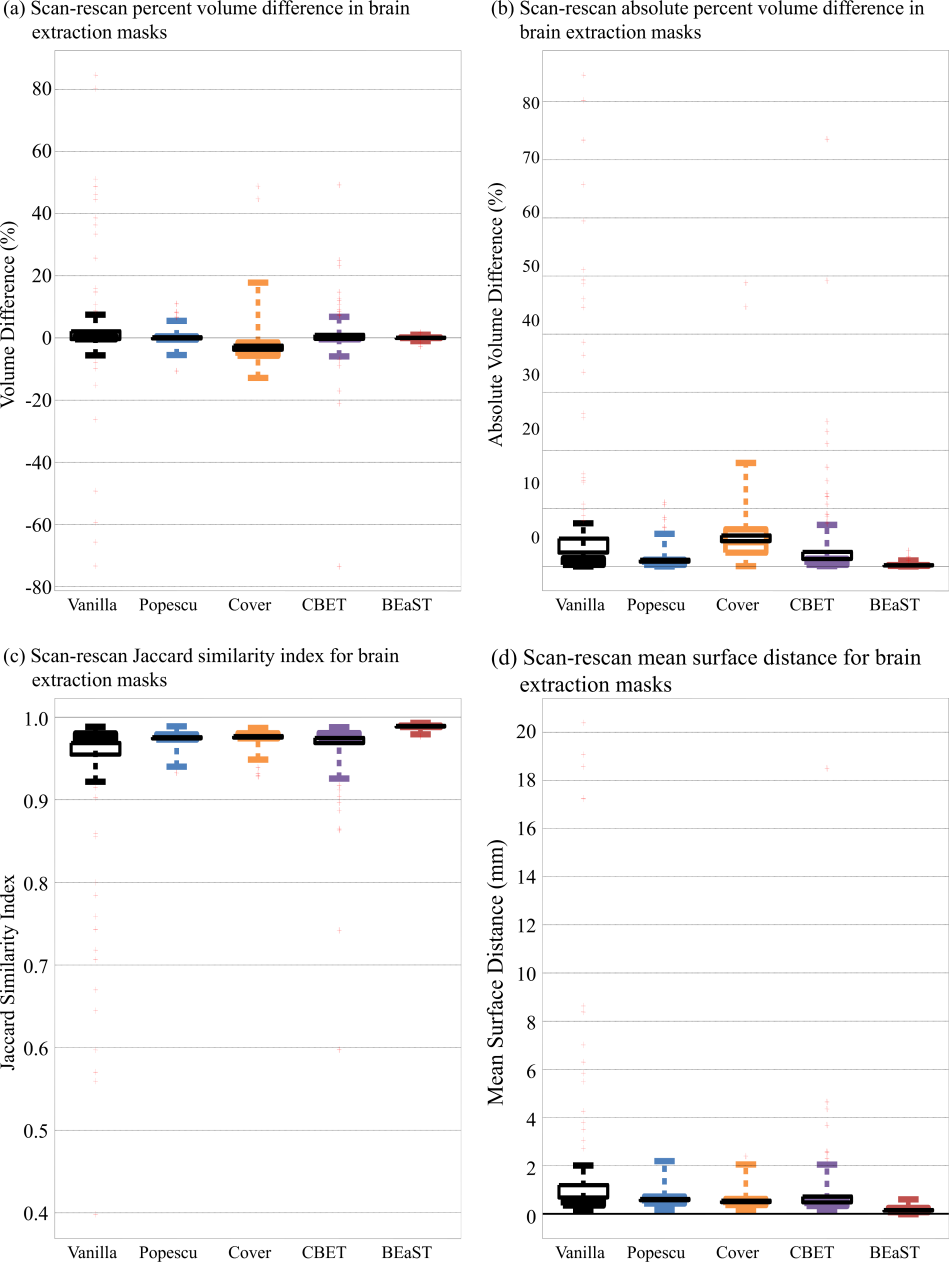
Scan-rescan comparison of brain extraction masks (Vanilla-BET, Popescu-BET, Cover-BET, CBET, and BEaST) in terms of (a) percent volume difference, (b) absolute percent volume difference, (c) Jaccard similarity index, and (d) mean surface distance. The colored box plot shows extreme values and interquartile range; the red crosses show the outliers with 2.5 SD limit; and the black lines indicate mean and its 95% confidence interval.

**Table 2(A)** shows the comparison of scan-rescan brain extraction masks. BEaST performed better in all the comparisons. Note for all measures (sensitivity, specificity, Jaccard index, and mean surface distance), the worst quartile from BEaST outperformed the best quartile of other methods. Note also that the volume difference in Fig 3A and the absolute volume difference in Fig 3B contain no outliers for BEaST, which is not the case for the other methods. There are few very low specificity cases in *Vanilla-SIENA* (n = 3) and *CBET-SIENA* (n = 1), and thus the means are similar to the 25-percentile value. **Table 2B, 2C and 2D** shows scan-rescan percent brain volume difference and absolute values from SIENA. *BEaST-SIENA* had the smallest standard deviation in scan-rescan percent brain volume difference among the 5 methods. The smallest mean and median absolute values were obtained by *BEaST-SIENA*, followed by *CBET-SIENA*, and *Cover-SIENA*. The absolute scan-rescan percent difference for *BEaST-SIENA* was significantly different from all other versions of SIENA (*p* < 0.0187, paired tests).

**Table 2:** Scan-rescan percent brain volume difference, number of failures, and scan-rescan percent brain volume difference.

**(a) Scan-Rescan Brain Extracted Mask Comparisons**					
	Vanilla-BET	Popescu-BET	Cover-BET	CBET-BET	BEaST
Percent Volume Difference (SD)	0.9 (11.7) %	0.0 (1.7) %	-3.1 (5.8) %	0.3 (5.9) %	0.0 (0.3) %
Percent Absolute Volume Difference (SD)	3.6 (11.2) %	1.0 (1.4) %	4.8 (4.4) %	1.9 (5.6) %	0.2 (0.3) %
Sensitivity (P25,P75)	0.961 (0.971,0.981)	0.975 (0.973,0.981)	0.976 (0.975,0.982)	0.971 (0.972,0.982)	0.989 (0.988,0.990)
Specificity (P25,P75)	0.995 (0.996,0.998)	0.994 (0.993,0.995)	0.995 (0.994,0.996)	0.996 (0.996,0.998)	0.999 (0.998,0.999)
Jaccard index (P25,P75)	0.962 (0.971,0.980)	0.975 (0.973,0.979)	0.976 (0.974,0.980)	0.972 (0.972,0.981)	0.989 (0.988,0.990)
Mean Surface Distance (P25,P75), mm	1.39 (0.79,1.06)	1.04 (0.87,1.16)	0.97 (0.81,1.07)	1.05 (0.78,1.04)	0.6 (0.52,0.70)
**(b) SIENA Robustness**					
	Vanilla-SIENA	Popescu-SIENA	Cover-SIENA	CBET-SIENA	BEaST-SIENA
#Failure, n (%)	2 (0.6%)	0 (0%)	0 (0%)	1 (0.3%)	0 (0%)
**(c) Scan-Rescan Percent Brain Volume Difference from SIENA**					
	Vanilla-SIENA	Popescu-SIENA	Cover-SIENA	CBET-SIENA	BEaST-SIENA
Mean (SD), %	0.036 (0.548)	-0.007 (0.533)	0.030 (0.535)	0.026 (0.481)	0.038 (0.473)
Median (P25, P75), %	0.038 (-0.166, 0.255)	0.017 (-0.210, 0.209)	0.055 (-0.134, 0.220)	0.039 (-0.153, 0.192)	0.045 (-0.118, 0.186)
**(d) Scan-Rescan Absolute Percent Brain Volume Difference from SIENA**					
	Vanilla-SIENA	Popescu-SIENA	Cover-SIENA	CBET-SIENA	BEaST-SIENA
Mean (SD), %	0.363 (0.406)	0.352 (0.396)	0.333 (0.417)	0.298 (0.372)	0.291 (0.368)
Median (P25, P75), %	0.202 (0.089, 0.514)	0.209 (0.098, 0.467)	0.181 (0.083, 0.408)	0.166 (0.078, 0.360)	0.154 (0.079, 0.344)
P-value†	<0.0001	<0.0001	0.0018	0.0187	-

P25, P75: 25th percentile and 75th percentile.

† Paired t-test with BEaST-SIENA.

**Table 3** shows the effect size for all methods estimated from the ADNI and OASIS datasets. The actual mean (SD) interval between the baseline and follow-up of 1.08 (0.07) for ADNI and 1.82 (0.40) for OASIS. *BEaST-SIENA* had the largest effect size: 41% and 27% larger, respectively for ADNI and OASIS, compared to *Vanilla-SIENA*.

**Table 3:** Mean (SD) annualized percent brain volume change (PBVC), effect size, number of failures, and required sample size per arm for each method and dataset.

**(a) ADNI**	** **	** **	** **	** **	** **
	Vanilla	Popescu	Cover	CBET	BEaST
Mean (SD) Normal PBVC, %	-0.651 (0.919)	-0.587 (0.909)	-0.561 (0.832)	-0.604 (0.798)	-0.599 (0.771)
Mean (SD) AD PBVC, %	-1.732 (1.478)	-1.747 (1.242)	-1.862 (1.304)	-1.832 (1.159)	-1.812 (1.150)
Effect Size	0.910	1.092	1.233	1.271	1.279
# Failures, n	5 (1.5%)	0 (0%)	0 (0%)	1 (0.3%)	0 (0%)
Required Sample Size per Arm†, n	310	212	167	158	155
BEaST Improvement in Sample Size (%)	50	27	7	2	-
**(b) OASIS**	** **	** **	** **	** **	** **
	Vanilla	Popescu	Cover	CBET	BEaST
Mean (SD) Nondemented, %	-0.463 (0.525)	-0.330 (0.692)	-0.413 (0.414)	-0.442 (0.436)	-0.414 (0.398)
Mean (SD) Demented, %	-1.275 (1.249)	-1.151 (1.383)	-1.178 (0.930)	-1.312 (1.236)	-1.191 (0.935)
Effect Size	0.860	0.760	1.078	0.924	1.095
# Failures, n	0	0	0	0	0
Required Sample Size per Arm†, n	341	436	218	296	210
BeaST Improvement in Sample Size (%)	37	52	4	29	-

† Sample size based on 80% power, 25% treatment effect, and 0.05-significance level and accounting for normal aging and failure rates.

Longitudinal scan-rescan experiment showed that *BEaST-SIENA* had the smallest mean absolute difference (0.11%) compared to other methods, best correlated with original PBVC values with the regression line closest to slope of 1 and intercept of 0 (Table 4). The effect size determined from the repeat dataset was overall lower than those calculated from the original PBVC but the trend that *BEaST-SIENA* performed optimally with *CBET-SIENA* closely next was similar to the scan-dataset.

**Table 4:** Repeat PBVC results and its comparison to original PBVC.

**(a) Mean (SD) percent brain volume change (PBVC), effect size, number of failures, and required sample size per arm for each method from repeat pairs in ADNI.**					
	Vanilla	Popescu	Cover	CBET	BEaST
Mean (SD) Normal PBVC, %	-0.712 (0.928)	-0.688 (0.888)	-0.631 (0.822)	-0.657 (0.782)	-0.648 (0.766)
Mean (SD) AD PBVC, %	-1.724 (1.405)	-1.708 (1.285)	-1.797 (1.357)	-1.762 (1.167)	-1.742 (1.162)
Effect Size	0.879	0.953	1.084	1.145	1.153
#Failures, n (%)	9 (2.8%)	7 (2.2%)	0 (0%)	2 (0.6%)	0 (0%)
Sample Size	335	283	214	192	190
**(b) Comparison of original PBVC and repeat PBVC in ADNI data.**					
Mean Difference in PBVC, %	-0.02	-0.03	-0.02	-0.01	-0.01
Mean Absolute Difference in PBVC, %	0.21	0.30	0.23	0.12	0.11
Correlation between original and repeat PBVC	0.922	0.915	0.950	0.968	0.971
Slope between original and repeat PBVC	0.900	0.906	0.948	0.953	0.959
Intercept between original and repeat PBVC	-0.134	-0.136	-0.076	-0.061	-0.055

PBVC: percent brain volume change

## Discussion and conclusion

Our study not only compared our results with those obtained using the default options of SIENA, but also with previously published “optimized” parameters [19, 20]. We have shown the improved reproducibility of BEaST brain extraction mask over the BET-derived brain masks in the scan-rescan dataset (Table 2, Fig 2 bottom and Fig 3), and better longitudinal consistency in the ADNI and OASIS datasets (Fig 2 top). The study also demonstrates the robust impact of BEaST brain masks on the measurement of whole brain atrophy using SIENA in ADNI and OASIS datasets (Tables 3 and 4).

The cross-sectional scan-rescan brain extraction comparisons in Fig 3 and Table 2 show that BEaST’s reproducibility is higher than the variants of BET studied here. Both BEaST and Popescu-BET masks were the least biased with a percent volume difference of <0.1%, However at 0.3%, the standard deviation of BEaST percent volume difference was almost 5 times smaller than Popescu-BET (1.4%), indicating more reproducible and robust brain masks for BEaST. Cover-BET performed worse, with a bias of -3.1% and a standard deviation of 5.8% for percent volume difference in the scan-rescan tests. For sensitivity and Jaccard index, the lowest 25 percentile of BEaST values was greater than the each of the BET variant 75 percentile values.

Better brain masks are likely to translate to improved scan-rescan SIENA measurements. Table 2C shows that all methods yield similar numbers with Popescu-SIENA giving the smallest bias for scan-rescan SIENA measurements. The absolute PBVC is also very similar between techniques, with *CBET-SIENA* and *BEaST-SIENA* yielding the smallest standard deviations.

A larger effect size means that smaller sample sizes are required to show a statistically significant difference between groups. The methods that consistently increased the effect size compared to *Vanilla-SIENA* were *Cover-SIENA*, *CBET-SIENA* and *BEaST-SIENA*, with the largest effect sizes achieved by *BEaST-SIENA* in both ADNI and OASIS datasets. *BEaST-SIENA* had 41% larger effect size than *Vanilla-SIENA* in ADNI data. *BEaST-SIENA* and Cover-Siena were robust without any processing failures, while the three other BET-derived SIENA pipelines failed to compute PBVC for some datasets in the ADNI cohort. There were no failures in the OASIS cohort. *Cover-SIENA*, *CBET-SIENA* and *BEaST-SIENA* result in the smallest required sample sizes with *CBET-SIENA* and *BEaST-SIENA* yielding almost the same sample size in the ADNI cohort, and *Cover-SIENA* and *BEaST-SIENA* yielding almost the same sample size in the OASIS cohort.

Our study did not evaluate the accuracy of PBVC measurements. The accuracy of BEaST has been studied in the original development and validation manuscript [6]. Creation of a gold standard for PBVC measurements is extremely difficult [22] as it requires manual segmentation of the brain parenchyma by multiple experts in high-resolution baseline and follow-up MRIs. Given the potential inter- and intra-rater variability in manual segmentation, it is not clear that such a gold standard would be good enough to quantify the differences between PBVC measures since the manual variability could be of the same magnitude as the errors and inaccuracies of the SIENA procedures using either BEaST- or BET-derived masks.

We did not investigate the effect of parameters directly used in SIENA. Since brain extraction is one the first processing steps in the SIENA pipeline, we expected the choice of brain mask would have the largest influence on the outcome of PBVC. We also did not investigate the sensitivity of templates in the BEaST library, as this was previously studied, and the use of n = 20 preselected templates appear to be sufficiently accurate [6]. We also did not assess the brain masks individually, reject results, or re-analyze data, which may take place in real analyses and in clinical trials. However, the robustness demonstrated by the scan-rescan measures (sensitivity, specificity, Jaccard, and means surface distance), average mask, the average difference mask, and greater statistical power suggests that such individual assessment may not be necessary with the *BEaST-SIENA* method.

Our results also highlight the variable performance of SIENA when BET is used for brain extraction. The published optimized method (*Cover-SIENA*) performed well and had similar effect sizes compared to the *BEaST-SIENA* pipeline in both ADNI and OASIS datasets. This high quality performance may be attributed to the fact that SIENA combines two masks (with an OR-operation) before brain tissue segmentation, thus reducing the risk of failure in case one brain mask is partially missing brain tissue. We have found BEaST to be very robust on over 3,000 datasets in other combined studies [9, 23–26].

The improved sample size estimation using *BEaST-SIENA* compared to the second best method (*Cover-SIENA*) was not large for ADNI dataset (7%, Table 3). However, our results show that SIENA results are very sensitive to BET options as changing the option “-f” from 0.1 to 0.2 changed the mean PBVC in the OASIS dataset by 25%, i.e., from -0.330 to -0.413 in non-demented group. Previous studies have attempted to optimize the parameters for BET in multiple sclerosis and AD studies [18–20] and resulted in varying parameters. Therefore, it may be difficult in prospective multi-center studies to predict optimal parameters for BET for both accuracy and effect size. The ranking of algorithms differed in BET-based SIENA pipelines between the ADNI and OASIS datasets, which may reflect the sensitivity of SIENA to brain extraction and emphasize its importance.

The ADNI standard analysis list contains 195 normal subjects and 133 AD patients for Complete 1 Year Visits [27]. Our cohort does not exactly match this list; one normal subject was removed due to poor quality, one normal subject is no longer available as of March 16, 2015, and 6 AD patients were added because Month-6 scans were missing while Screening and Year-1 were available.

In conclusion, BEaST was robust in two large studies (ADNI and OASIS), consistent across and within subjects, and led to more reproducible and sensitive brain volume change measurements than conventional BET when applied in SIENA.

## Supporting information

S1 File Scan-rescan results. (XLSX)

S2 File SIENA results for ADNI and OASIS. (XLSX)
